# Diversity, Antibiotic Resistance, and Biofilm-Forming Ability of Enterobacteria Isolated from Red Meat and Poultry Preparations

**DOI:** 10.3390/microorganisms8081226

**Published:** 2020-08-12

**Authors:** Rosa Capita, Ana Castaño-Arriba, Cristina Rodríguez-Melcón, Gilberto Igrejas, Patricia Poeta, Carlos Alonso-Calleja

**Affiliations:** 1 Department of Food Hygiene and Technology, Veterinary Faculty, University of León, E-24071 León, Spain; crodm@unileon.es (C.R.-M.); carlos.alonso.calleja@unileon.es (C.A.-C.); 2 Institute of Food Science and Technology, University of León, E-24071 León, Spain; acasa@unileon.es; 3 Associated Laboratory for Green Chemistry, University NOVA of Lisboa, 2829-516 Caparica, Portugal; gigrejas@utad.pt (G.I.); ppoeta@utad.pt (P.P.); 4 Department of Genetics and Biotechnology, University of Trás-os-Montes and Alto Douro, 5000-811 Vila Real, Portugal; 5 Functional Genomics and Proteomics Unit, University of Trás-os-Montes and Alto Douro, 5000-811 Vila Real, Portugal; 6 Department of Veterinary Sciences, University of Trás-os-Montes and Alto Douro, 5000-811 Vila Real, Portugal

**Keywords:** meat preparations, enterobacterial species, antibiotic resistance, biofilm-forming ability

## Abstract

A total of 44 samples of beef, pork, and poultry preparations were tested. Average counts (log cfu/g) of enterobacteria were 1.99 ± 0.99 (beef preparations), 1.96 ± 1.44 (pork), 2.09 ± 0.92 (chicken), and 2.17 ± 1.06 (turkey) (*p* > 0.05). Two hundred enterobacterial strains were identified and 13 genera (21 species) were distinguished, including species that are a significant cause of infection. The most common genera were *Escherichia* (32.5% of strains), *Serratia* (17.0%), *Hafnia* (12.5%), and *Salmonella* (12.0%). Isolates were screened by disc diffusion for susceptibility to 15 antibiotics. A total of 126 strains (63% of the isolates) were multirresistant (having resistance to two or more antibiotics), 46 (23%) were resistant to one antibiotic, and 28 (14%) were sensitive to all antibiotics. The average number of resistances per strain was 2.53 ± 2.05. A higher (*p* < 0.05) average number of resistances was observed in strains from turkey (3.14 ± 2.55) than in strains from beef (2.15 ± 1.22), pork (2.16 ± 1.39), or chicken (2.44 ± 2.22). At least 50% of strains showed resistance or reduced susceptibility to ampicillin, cefotaxime, ceftazidime, or streptomycin, considered to be “critically important” antimicrobial agents in human medicine. Seventy-nine strains (39.5%), 60 strains (30.0%), and 46 strains (23.0%) were weak, moderate, and strong biofilm producers (crystal violet assay), respectively. This investigation provides evidence that bacteria from red meat and poultry preparations pose major potential risk to consumers.

## 1. Introduction

The world per capita consumption of the three most consumed types of meat in 2017 was 15.7, 15.2, and 9.0 kg per person per year, for pork, poultry, and beef, respectively [1]. A certain percentage of meat is consumed in the form of meat preparations. Regulation (EC) 853/2004 defines meat preparations as fresh meat, including meat that has been reduced to fragments, which has had foodstuffs, seasonings or additives added to it or which has undergone processes insufficient to modify the internal muscle fibre structure of the meat and thus to eliminate the characteristics of fresh meat [2].

The extensive consumption of red meat and poultry leads to concerns that the products marketed should be safe, have a low spoilage rate, and show the right composition, packaging, colour, taste, and appearance. In such a scenario, products excessively contaminated with microorganisms are undesirable [3]. Enterobacteria are interesting to evaluate for meat because they act as indicators of its microbiological quality and the level of hygiene in the processes of production and handling, in addition to helping to predict the potential shelf-life of products [4,5]. Moreover, the family *Enterobacteriaceae* includes several species that are a significant cause of infection in both community and nosocomial settings [6].

There have been increasing serious concerns about bacterial resistance to drugs at both national and international levels. Antimicrobial resistance has been defined as a global pandemic, one of the major global public health threats, and consequently one of the 21st century’s major health challenges [7]. Antibiotic resistance is on a rising trend, with estimates that in three decades’ time, infections by antibiotic-resistant bacteria will be the main cause of world mortality at ten million deaths each year worldwide. These figures should be contrasted with the 700,000 deaths attributable to antibiotic resistance in 2014 [8]. The financial consequences of resistance to antibiotics are also of considerable weight, with estimates that these infections cost the health care systems of EU and EEA countries 1.1 thousand million euros each year [9].

The presence of antibiotic-resistant bacteria in foods is a direct risk for consumers due to the potential of these microorganisms to cause hard-to-treat foodborne infections. There is also an indirect risk of the horizontal transfer of resistance genes to pathogenic microorganisms, including among unrelated genera, at various points along the food chain [7]. One enterobacteria species, *Escherichia coli*, acts as reservoir of resistance genes, which is a worrying fact in the context of public health since it means there is a high likelihood of gene transfer to other, pathogenic, bacteria. Moreover, this circumstance enables this bacterial group to be used as sentinel for resistance to antibiotics [10]. Monitoring resistance to antibiotics is essential not only to obtain information about the magnitude of this problem and trends within it, but also to plan and monitor the effectiveness of any control measures introduced.

Biofilms are the predominant mode of microbial growth in nature [11]. These structures are defined as complex communities of microorganisms embedded in an extracellular polymer matrix synthesized by the microorganisms themselves, with the ability to adhere to a variety of different biotic or abiotic surfaces [12,13]. As regards the food industry, once biofilms become established, the resident bacteria display enhanced resistance to different environmental stresses, thus encouraging their persistence over long periods and increasing the risk of contamination of foodstuffs [14]. Biofilms present on equipment and installation surfaces in the food industry have been identified as the cause of the greater part of outbreaks of food-borne disease [15].

There is extremely limited information about species, resistance to antibiotics and biofilm production in enterobacteria from meat in north-west Spain. The aim of the present work was to gain awareness of the patterns of antibiotic resistance and the biofilm-forming ability of enterobacterial species isolated from red meat- and poultry-based preparations.

## 2. Materials and Methods

### 2.1. Samples

A total of 44 raw sample preparations weighing approximately 250 g each were acquired from different supermarkets in the city of León in north-west Spain. The beef preparations were hamburgers (10 samples); the pork preparations included meatballs (2), minced meat (6), hamburgers (2), and sausages (4); the chicken preparations included hamburgers (4), nuggets (2), and sausages (2); and the turkey preparations included meatballs (4) and hamburgers (8). Each sample was placed in a separate sterile plastic bag, transported to the laboratory immediately in an ice chest, and tested upon arrival or stored at 3 ± 1 °C for no longer than 4 h prior to starting the analyses.

### 2.2. Microbiological Analysis

The samples, each weighing 10 g, were placed in a sterile stomacher bag containing 90 mL of sterile 0.1% (wt/vol) peptone water (Oxoid Ltd., Basingstoke, England) and homogenized (Masticator IUL, Barcelona, Spain) for 2 min. The 10 g samples were taken from two or more pieces of meat in the same lot. Serial decimal dilutions in the same diluent were prepared from the homogenate. Duplicate pour plates of violet red bile glucose agar (VRBGA; Oxoid) with overlay, prepared using 1 mL volumes of appropriate dilutions, were incubated at 37 °C for enterobacteria enumeration. Plates with typical colonies were counted, and mean counts were calculated. Microbial counts were transformed to log_10_ cfu/g.

### 2.3. Species Identification

Three to eight colonies in VRBGA were selected from the plates of each sample, transferred onto tryptone soy agar (TSA; Oxoid), and incubated at 37 °C for 24 h to obtain pure cultures, which were examined for colony and cell morphology, Gram stain, and oxidase and catalase activities. Strains corresponding to Gram-negative, catalase-positive, and oxidase-negative bacilli were identified with the aid of API 20E strips (bioMérieux, Marcy L’Étoile, France) in accordance with the manufacturer’s instructions. Data interpretation was carried out using the Analytical Profile Index (API) database (V5.0) with apiweb™ identification software (bioMérieux). The strains were kept frozen at −50 °C after re-suspension in tryptone soy broth (TSB; Oxoid) with 20% (vol/vol) glycerol.

### 2.4. Antimicrobial Susceptibility Testing

A total of 200 isolates were screened on Mueller Hinton agar (Oxoid) for susceptibility to a panel of fifteen antibiotics, using the disc-diffusion method as described in the Clinical and Laboratory Standards Institute (CLSI) guidelines [16]. The following discs (Oxoid) were used: amoxicillin-clavulanic acid (AMC, 30 μg), ampicillin (AMP, 10 μg), aztreonam (ATM, 30 μg), cefotaxime (CTX, 30 μg), cefoxitin (FOX, 30 μg), ceftazidime (CAZ, 30 μg), chloramphenicol (C, 30 μg), ciprofloxacin (CIP, 5 μg), amikacin (AK, 30 μg), gentamicin (CN, 10 μg), streptomycin (STR, 10 μg), imipenem (IMP, 10 μg), nalidixic acid (NA, 30 μg), trimethoprim/sulfamethoxazole (SXT, 25 μg), and tetracycline (TE, 30 μg). After incubation at 37 °C for 18 to 24 h, the inhibition halos were measured and scored as susceptible, intermediate (reduced susceptibility), or resistant. *Escherichia coli* ATCC 25922 and *Staphylococcus aureus* ATCC 29213 were used as the reference strains for antibiotic disc control.

### 2.5. Biofilm Determination

A previously described procedure [11] was followed to quantify the biofilms. Strains (200) cultured on TSA were transferred to TSB and incubated at 37 °C for 18 h. Once this time had elapsed, the tubes held a concentration of approximately 10^9^ cfu/mL. Four decimal dilutions in TSB were performed to yield concentrations of 10^5^ cfu/mL, which were then used to inoculate the wells of polystyrene microtitre plates (Oy Growth Curves Ab Ltd., Helsinki, Finland). The wells were filled with 225 µL of TSB and 25 µL of bacterial culture, so that the final concentration in the well was 10^4^ cfu/mL. Negative controls were included, containing 250 µL of TSB. After incubation at 37 °C for 24 h, the content of the plate was poured off and the wells washed with 300 µL of sterilized distilled water. The bacteria that remained attached were fixed by adding 250 µL of methanol to each well for 15 min. The plates were then emptied, air dried, and stained for five minutes with 250 µL per well of an aqueous solution of 0.5% crystal violet. The wells were then emptied and washed by placing the plate under running tap water. The plates were subsequently air dried, and the dye bound to the adherent cells was re-solubilized with 250 µL of 33% acetic acid (Sigma-Aldrich Co., St. Louis, MO, USA) per well, the substance being allowed to work for one minute. Optical density at 580 nm (OD_580_) was determined in a Bioscreen C MBR (Oy Growth Curves Ab). The micro-well plates were agitated for one minute prior to measuring the turbidity. Control strains that were strong and weak formers of biofilm from the culture collection of the University of León, Spain, were included in each experiment.

The strains were classified as a function of their capacity to form biofilms. The cut-off OD_580_ (ODc) was defined as three standard deviations above the mean OD_580_ of the negative controls. The strains were split into four categories: not biofilm producers, when OD_580_ ≤ ODc; weak biofilm producers, when ODc < OD_580_ ≤ (2ODc); moderate biofilm producers, when (2ODc) < OD_580_ ≤ (4ODc); and strong biofilm producers, when (4ODc) < OD_580_ [11].

### 2.6. Statistical Analysis

The microbial counts (log_10_ cfu/g) and differences in the extent of biofilm formation were examined by analysis of variance (ANOVA) techniques, using Duncan’s multiple range test to separate averages. The prevalence of resistance in different enterobacterial species was compared using Fisher’s Exact Test. Significance was determined at the 95% (*p* < 0.05) level. All the tests were carried out using the Statistica^®^ 8.0 package (Statsoft Ltd., Tulsa, OK, USA).

## 3. Results and Discussion

### 3.1. Enterobacteria Load and Identification

All the meat and poultry preparation samples harboured enterobacteria. Average enterobacteria loads of 2.04 ± 1.12 log cfu/g were obtained. Similar (*p* > 0.05) counts (log_10_ cfu/g) were observed for beef (1.99 ± 0.99), pork (1.96 ± 1.44), chicken (2.09 ± 0.92), and turkey (2.17 ± 1.06) preparations.

Most of the enterobacteria found in meat come from contamination with feces due to bowel rupture or use of contaminated water during slaughtering and evisceration. Their presence in large numbers may therefore indicate poor hygiene in the slaughterhouse from which the meat is sourced, insufficiently hygienic handling, inappropriate storage, or a combination of these [17,18,19]. It should be noted that all the counts for these microbial groups fulfilled the guideline microbiological criteria, which state that they should not exceed 2 (maximum 4) log units/g [20,21] or 3.5 (maximum 4.5) log units/g [22]. Similar counts to those in the present study regarding the presence of enterobacteria in meat have previously been found [23,24] and observed by other authors [25].

Two hundred enterobacteria were isolated from beef (47), pork (32), chicken (64), and turkey (57) preparations. A total of 24 *S. enterica* strains were detected (12.0% of the 200 isolates) (Table 1). The prevalence of *S. enterica* was 8.5%, 31.3%, 7.8%, and 8.8% for beef, pork, chicken, and turkey, respectively. These prevalence values are within the range of the data obtained in other research works for red meat and poultry, where values of 3.6% [26], 9.0% [27], 12.4% [28], 18.1% [29], and 34.0% [30] have been reported. It should be noted, however, that no enrichment steps were used for the *Salmonella* isolation in the present study, which could have influenced the results obtained.

Fifty-five bacterial isolates out of 200 (27.5%) were identified as *E. coli*. This bacterium has been isolated in a high percentage from raw meat and unprocessed ready-to-eat products in several studies [31,32]. The rest of strains detected belonged to the species *Escherichia vulneris* (10 strains; 5%), and to the genera *Serratia* (34; 17.0%), *Hafnia* (25; 12.5%), *Enterobacter* (19; 9.5%), *Klebsiella* (14; 7.0%), *Pantoea* (5; 2.5%), *Yersinia* (4; 2.0%), *Proteus* (3; 1.5%), *Citrobacter* (2; 1.0%), *Kluyvera* (2; 1.0%), *Providencia* (2; 1.0%), and *Cedecea* (1; 0.5%) (Table 1). These genera have been previously detected in meat [32,33]. The number of different bacterial species isolated from each type of meat preparation ranged from 8 (turkey) to 13 (pork and chicken). The most frequent species in each type of meat preparation were *E. coli* (chicken and turkey), *Hafnia alvei* (beef and pork), *S. enterica* (pork), and *Serratia liquefaciens* (beef and turkey). Notably, the majority of species detected in red meat and poultry preparations have been implicated in human disease, for instance *S. enterica*, *E. coli*, *H. alvei, Klebsiella pneumoniae*, *Kluyvera* spp., and *S. liquefaciens* [34]. It should be noted that these results should be considered with caution, because phenotypic methods may lead to misidentification in some cases [35].

### 3.2. Antimicrobial Susceptibility

Two hundred enterobacteria isolates from red meat and poultry preparations were screened for susceptibility to 15 antibiotics. Table 2 shows the number and percentage of strains that were multirresistant (having resistance to two or more antibiotics; M), resistant to one antibiotic (R), and sensitive to all antibiotics (S). A total of 126 (63%), 46 (23%), and 28 (14%) strains were classified as M, R or S, respectively. Multirresistant strains were resistant to 2 (43 strains), 3 (28), 4 (25), 5 (12), 6 (6), 7 (5), 8 (5), or 9 (2) antibiotics. The considerable prevalence of resistant and multirresistant strains observed in the work being reported here is worrying given that the resistances detected would probably undermine the usefulness as a therapeutic option of several antibiotics used in both human and veterinary medicine. Infections caused by multirresistant bacteria are not only associated with high morbidity and mortality rates, but also with increased treatment costs [36,37].

The average number of resistances per strain was 2.53 ± 2.05. A higher (*p* < 0.05) average number of resistances was observed in strains from turkey (3.14 ± 2.55) than in strains from beef (2.15 ± 1.22), pork (2.16 ± 1.39), or chicken (2.44 ± 2.22). These average values are in the range of those previously recorded for Gram-negative bacteria in meat, with 0.57 [23], 0.78 [37], 3.76 [31], and 4.0 [38] antimicrobial resistances found for enterobacteria isolates from meat. It should be noted that the highest average number of antibiotic resistances was shown by *Enterobacter amnigenus* (4.75 ± 0.50) and *E. coli* (3.95 ± 2.46). This fact is a matter of concern because both bacterial species are frequent human pathogens [37,39]. By contrast, *Serratia plymuthica* (0.00 ± 0.00), *Citrobacter freundii* (1.00 ± 0.00), and *Providencia alcalifaciens* (1.00 ± 1.41) had the lowest number of antibiotic resistances (Table 1).

**Figure 1:**
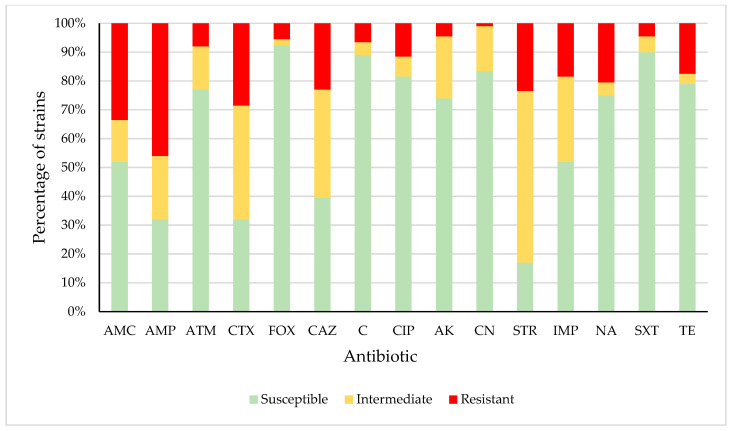
Percentage of *Enterobacteriaceae* strains susceptible, intermediate, or resistant to each antibiotic tested. Amoxicillin-clavulanic acid (AMC), ampicillin (AMP), aztreonam (ATM), cefotaxime (CTX), cefoxitin (FOX), ceftazidime (CAZ), chloramphenicol (C), ciprofloxacin (CIP), amikacin (AK), gentamicin (CN), streptomycin (STR), imipenem (IMP), nalidixic acid (NA), trimethoprim/sulfamethoxazole (SXT), and tetracycline (TE).

Figure 1 shows the percentage of strains that were susceptible, intermediate, or resistant to each of the antibiotics tested. Resistance was observed in enterobacteria isolates relating to amoxicillin/clavulanic acid (AMC) (33.5% of strains), ampicillin (AMP) (46.0%), aztreonam (ATM) (8.0%), cefotaxime (CTX) (28.5%), cefoxitin (FOX) (5.5%), ceftazidime (CAZ) (23.0%), chloramphenicol (C) (6.5%), ciprofloxacin (CIP) (11.5%), amikacin (AK) (4.5), gentamicin (CN) (1.0%), streptomycin (STR) (23.5%), imipenem (IMP) (18.5%), nalidixic acid (NA) (20.5%), trimethoprim/sulfamethoxazole (SXT) (4.5%), and tetracycline (TE) (17.5%). Resistance or reduced susceptibility was observed in the case of AMC (48.0% of strains), AMP (68.0%), ATM (23.0%), CTX (68.0%), FOX (7.5%), CAZ (60.5%), C (11.0%), CIP (18.5%), AK (26.0), CN (16.5%), STR (83.0%), IMP (48.0%), NA (25.0%), SXT (10.0%), and TE (21.0%). High levels of resistance to such antimicrobials have also been reported previously in enterobacteria isolated from red meat and poultry [37,40,41,42,43,44,45].

The great number of resistant strains in foods of animal origin observed in different research works appears to be related to the use of antibiotics in animal production and clinical practice [7]. In the present study, a considerable prevalence of resistance to antibiotics widely used in animal production was observed [46,47,48]. Notably, however, a high prevalence of resistance was also observed for substances whose use has been prohibited in food-producing animals for some decades, for instance, chloramphenicol. Mechanisms of cross-resistance and co-resistance may have contributed to the persistence over time of genes for resistance to these substances, as has previously been suggested [7,41].

It should be noted that at least 50% of the strains showed resistance or reduced susceptibility to ampicillin, cefotaxime, ceftazidime, or streptomycin. These four compounds are considered as ‘‘critically important’’ antimicrobial agents in human medicine, according to the World Health Organisation [49]. The World Organisation of Animal Health [50] lists ampicillin and streptomycin as “veterinary critically important antimicrobial agents”.

Eighty-eight antibiotic resistance patterns were observed among the 200 enterobacterial strains. Eight patterns stand out as particularly frequent: AMC/AMP (showed by 12 strains; 6.0%), AMP (10 strains; 5.0%), CTX/IMP (9 strains; 4.5%), AMC/AMP/CAZ (7 strains; 3.5%), NA (7 strains; 3.5%), IMP (6 strains; 3.0%), AMC (5 strains; 2.5%), and CTX (5 strains; 2.5%).

### 3.3. Biofilm Production

Biofilm production facilitates bacterial persistence in food processing facilities, providing a favourable environment for the exchange of antibiotic resistance genes. A total of 185 (92.5%) of the strains examined here were able to form biofilm on polystyrene, with 79 (39.5%) isolates as weak, 60 (30.0%) isolates as moderate, and 46 (23.0%) isolates as strong biofilm producers. The mean OD_580_ (crystal violet assay) value for the 200 enterobacteria strains was 0.524 ± 0.581. The OD_580_ observed in the present investigation for not biofilm producer strains was 0.112 ± 0.004. The data for weak, moderate, and strong biofilm producers were 0.209 ± 0.071, 0.343 ± 0.041, and 0.767 ± 0.118, respectively (*p* < 0.05). The ability of enterobacteria to produce biofilms on polystyrene has previously been demonstrated [11,36]. These findings are a matter of concern because plastic materials are frequently used to manufacture a range of surfaces in food processing facilities, such as piping, cutting boards, and other equipment [11,36].

A lower (*p* < 0.05) average number of resistances was observed for not biofilm producer (1.80 ± 1.61) than for strong biofilm producer strains (2.91 ± 2.41). Weak and moderate biofilm producers were resistant to 2.53 ± 1.97 and 2.40 ± 1.94 antibiotics, respectively. Other studies have also shown that antibiotic resistance is greater among strains that are biofilm producers than among those that are not biofilm producers [51,52]. By contrast, several authors have observed no relationship between biofilm production and antibiotic resistance [53].

**Figure 2:**
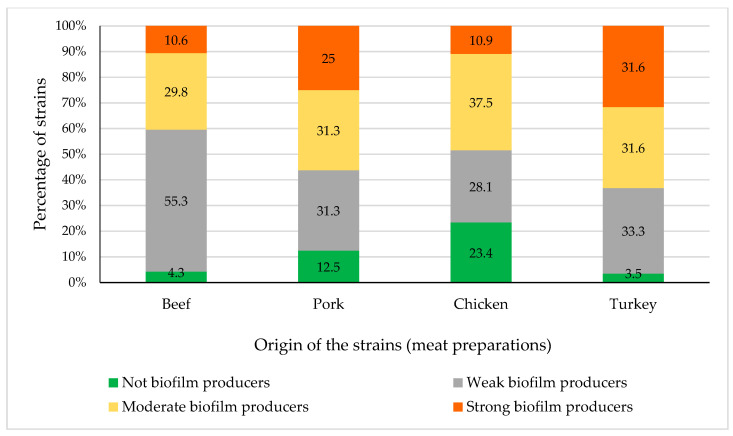
Percentage of not biofilm producer isolates and of weak, moderate, and strong biofilm producer isolates from beef, pork, chicken, and turkey preparations.

Figure 2 shows the percentage of not biofilm producer and weak, moderate, and strong biofilm producer isolates from beef, pork, chicken, and turkey preparations, respectively. No differences (*p* > 0.05) were observed in the biofilm-forming ability of enterobacteria isolated from preparations of different animal species.

Table 3 shows the biofilm-forming ability of each enterobacterial species isolated from poultry and meat preparations. The highest percentage of not biofilm producer strains was shown by *C. freundii* (100% of strains were not biofilm producers), *E. amnigenus* (25%), and *S. enterica* (21%). These results do not agree with previous findings, where all *S. enterica* strains were able to form biofilm [11,37]. These discrepancies among research works may be due to variations among different strains of the same species, or even of the same serotype, regarding their ability to form biofilm [11]. While the microtiter crystal violet assay is one of the most widely used methods for assessing biofilm formation, because it is a fast, high-throughput screening tool with great value for microbiologists, this assay is known for its substantial deviation from experiment to experiment, and even from well to well. The microtiter biofilm assay involves several pitfalls and can result in scattered results due to heterogeneous structured growth, disruptive procedures, and shortcomings in quantifying biomass and viable cells [54,55]. These facts could also be responsible for the differences observed between research works.

## 4. Conclusions

The red meat and poultry preparations harboured enterobacteria of pathogenic species, such as *S. enterica*. This fact suggests the risk of human infection through the consumption of raw or undercooked meat preparations and the risk of cross-contamination to other food products. A worrying fact emerging from this research is the considerable prevalence of resistance to antibiotics observed among the strains of enterobacteria isolated from red meat and poultry preparations. No substantial differences were observed between the levels or the prevalence of resistance to antibiotics in the bacteria isolated from the different meat preparations. The high percentage of strains able to form biofilm (92.5%) is also a matter of concern. The results of this study provide information on the prevalence of antibiotic resistance and biofilm-forming ability in enterobacteria isolates from red meat and poultry preparations in Spain.

## Figures and Tables

**Table 1:** Identification and origin (animal species) of 200 enterobacteria strains isolated from red meat and poultry preparations.

Enterobacterial Species	Origin (Number of Strains)				
	Beef (47)	Pork (32)	Chicken (64)	Turkey (57)	Average Number of Antibiotic Resistances
*Cedecea lapagei*			1		2.00 ± 0.00
*Citrobacter braakii*		1			2.00 ± 0.00
*Citrobacter freundii*		1			1.00 ± 0.00
*Enterobacter aerogenes*		1			2.00 ± 0.00
*Enterobacter amnigenus*		1	3		4.75 ± 0.50
*Enterobacter asburiae*	4	1		4	1.11 ± 1.36
*Enterobacter cloacae*		1	4		1.60 ± 0.55
*Escherichia coli*		1	25	29	3.95 ± 2.46
*Escherichia vulneris*	5		5		1.10 ± 0.32
*Hafnia alvei*	10	8	3	4	2.84 ± 1.07
*Klebsiella oxytoca*	4		5		2.11 ± 1.05
*Klebsiella pneumoniae*	1	2		2	2.00 ± 1.87
*Kluyvera* spp.	1		1		3.00 ± 1.41
*Pantoea* spp.	1		2	2	1.20 ± 1.64
*Proteus vulgaris*	3				1.67 ± 0.58
*Providencia alcalifaciens*	1		1		1.00 ± 1.41
*Salmonella enterica*	4	10	5	5	2.17 ± 1.66
*Serratia liquefaciens*	13	3	6	10	1.59 ± 1.64
*Serratia marcescens*		1			2.00 ± 0.00
*Serratia plymuthica*		1			0.00 ± 0.00
*Yersinia* spp.			3	1	2.25 ± 3.86

**Table 2:** Number and percentage of multirresistant (M), resistant (R), and sensitive (S) enterobacterial strains isolated from red meat and poultry preparations.

	M		R		S	
Animal Species	No	%	No	%	No	%
Beef (*n* = 47)	33	70%	10	21%	4	9%
Pork (*n* = 32)	21	66%	6	19%	5	16%
Chicken (*n* = 64)	35	55%	18	28%	11	17%
Turkey (*n* = 57)	37	65%	12	21%	8	14%
Total (*n* = 200)	126	63%	46	23%	28	14%

**Table 3:** Biofilm-forming ability of different bacterial species isolated from red meat and poultry preparations.

	Number of Strains (%)			
Bacterial Species	Not Biofilm Producer	WeakBiofilm Producer	ModerateBiofilm Producer	StrongBiofilm Producer
*Cedecea lapagei*		1 (100%)		
*Citrobacter braakii*		1 (100%)		
*Citrobacter freundii*	1 (100%)			
*Enterobacter aerogenes*				1 (100%)
*Enterobacter amnigenus*	1 (25%)			3 (75%)
*Enterobacter asburiae*		5 (56%)	3 (33%)	1 (11%)
*Enterobacter cloacae*			2 (40%)	3 (60%)
*Escherichia coli*	5 (9%)	23 (42%)	14 (25%)	13 (24%)
*Escherichia vulneris*	1 (10%)	5 (50%)	2 (20%)	2 (20%)
*Hafnia alvei*		9 (36%)	12 (48%)	4 (16%)
*Klebsiella oxytoca*		3 (33%)	4 (44%)	2 (22%)
*Klebsiella pneumoniae*			1 (20%)	4 (80%)
*Kluyvera* spp.		2 (100%)		
*Pantoea* spp.		4 (80%)	1 (20%)	
*Proteus vulgaris*		3 (100%)		
*Providencia alcalifaciens*		1 (50%)	1 (50%)	
*Salmonella enterica*	5 (21%)	5 (21%)	12 (50%)	2 (8%)
*Serratia liquefaciens*	2 (6%)	16 (50%)	5 (16%)	9 (28%)
*Serratia marcescens*				1 (100%)
*Serratia plymuthica*				1 (100%)
*Yersinia* spp.		1 (25%)	3 (75%)	
